# Isolating the Unique and Generic Movement Characteristics of Highly Trained Runners

**DOI:** 10.3390/s21217145

**Published:** 2021-10-28

**Authors:** Fabian Hoitz, Laura Fraeulin, Vinzenz von Tscharner, Daniela Ohlendorf, Benno M. Nigg, Christian Maurer-Grubinger

**Affiliations:** 1 Biomedical Engineering Graduate Program, Schulich School of Engineering, University of Calgary, 2500 University Drive NW, Calgary, AB T2N 1N4, Canada; nigg@ucalgary.ca; 2 Human Performance Laboratory, Faculty of Kinesiology, University of Calgary, 2500 University Drive NW, Calgary, AB T2N 1N4, Canada; tvvon@ucalgary.ca; 3 Institute for Occupational Medicine, Social Medicine and Environment Medicine, Goethe-University Frankfurt, Theodor-Stern-Kai 7, Building 9a, 60590 Frankfurt am Main, Germany; fraeulin@med.uni-frankfurt.de (L.F.); ohlendorf@med.uni-frankfurt.de (D.O.); maurer-grubinger@med.uni-frankfurt.de (C.M.-G.)

**Keywords:** running, triathlon, movement pattern, human recognition, artificial neural network, layer-wise relevance propagation, machine learning

## Abstract

Human movement patterns were shown to be as unique to individuals as their fingerprints. However, some movement characteristics are more important than other characteristics for machine learning algorithms to distinguish between individuals. Here, we explored the idea that movement patterns contain unique characteristics that differentiate between individuals and generic characteristics that do not differentiate between individuals. Layer-wise relevance propagation was applied to an artificial neural network that was trained to recognize 20 male triathletes based on their respective movement patterns to derive characteristics of high/low importance for human recognition. The similarity between movement patterns that were defined exclusively through characteristics of high/low importance was then evaluated for all participants in a pairwise fashion. We found that movement patterns of triathletes overlapped minimally when they were defined by variables that were very important for a neural network to distinguish between individuals. The movement patterns overlapped substantially when defined through less important characteristics. We concluded that the unique movement characteristics of elite runners were predominantly sagittal plane movements of the spine and lower extremities during mid-stance and mid-swing, while the generic movement characteristics were sagittal plane movements of the spine during early and late stance.

## 1. Introduction

Movement characteristics appear to be similar across individuals and/or functional groups. Movement patterns derived from males, for example, shared a more pronounced shoulder sway, while movement patterns derived from females shared a more pronounced hip sway [1,2]. However, it has also been demonstrated that movement patterns can be as unique to individuals as their fingerprints [3,4,5]. Pataky et al. [6], for instance, accurately identified 104 individuals based only on their plantar pressure distribution during walking. It appears, therefore, that certain characteristics of human movement are shared amongst individuals/functional groups while other movement characteristics appear to be unique.

Supporting this notion, our recent work highlighted that some movement characteristics (e.g., joint angles in the coronal and transverse plane) were highly important for the identification of a specific individual within a cohort of novice runners, while other movement characteristics (e.g., joint angles in the sagittal plane) were less important for said identification [7]. We speculate, therefore, that a movement pattern—defined through 3D joint angle trajectories—may be comprised of at least two types of movement characteristics: those that are specific to an individual (i.e., unique), and those that are common to multiple individuals (i.e., generic). Understanding unique and generic movement characteristics may be crucial for health- [8,9], security- [10], and performance-related [11] applications. A gait-based identification system that considers only unique movement characteristics, for example, may be more difficult to breach, and the generic movement characteristics of a population of elite marathon athletes may isolate biomechanically relevant aspects of an efficient running style.

By applying layer-wise relevance propagation (LRP) to a neural network that was trained to identify individuals based on their movement patterns, one may isolate unique and generic movement characteristics for a given population. LRP is an analysis method that addresses the ‘black-box’ nature [12] of neural networks and highlights why a certain decision was reached by a classifier [13]. It has been applied in image classifications [14], text document classifications [15] and, recently, in biomechanics [8,9]. Conceptually, LRP calculates a relevance score for each input variable of a neural network (i.e., each data point in a movement pattern). These relevance scores indicate how important a given input variable was for the decision reached by the model. High absolute relevance scores represent input variables that were crucial to the decision of the network while low absolute relevance scores (approx. 0) indicate those input variables that were less crucial to the final decision of the network.

In biomechanics, LRP has been applied by Aeles et al. [8], who used this method to highlight features of electromyograms that revealed unique muscle activation patterns while walking and pedaling. Horst et al. [9] used LRP to highlight individual-specific features of joint angle trajectories and ground reaction forces during barefoot walking. Lastly, we previously [7] used LRP to distinguish the characteristics of movement patterns that were very important for the identification of novice runners from those that were less important in order to minimize the amount of data needed for human identification. It is evident that LRP is gaining traction in the field of biomechanics and, consequently, it is crucial to gain a clear understanding of the functional meaning of relevance scores.

In the context of human recognition based on movement patterns, we propose that variables with high (absolute) relevance scores may encode unique characteristics of movement patterns, while variables with low relevance scores may encode the more generic features of movement patterns. Isolating and understanding the unique/generic features of movement patterns is valuable, especially within a population of elite runners. That is because the shared movement characteristics of elite level athletes (i.e., generic features) potentially highlight performance-related characteristics that may be essential to an economical running style. Consequently, aspiring professional runners should be encouraged to model their personal running style after the generic movement characteristics of elite runners. Unique aspects of elite level runners, on the other hand, may describe athlete-specific movement strategies; therefore, a deeper understanding of an athlete’s unique movement characteristics might inspire custom-tailored training interventions that maximize the potential of a given athlete. The unique and generic movement characteristics of elite runners, however, remain unknown.

Consequently, the purpose of this work was to isolate the unique and generic movement characteristics of elite-level triathletes and to understand if they are expressed by variables with high/low relevance scores derived from a neural network trained to recognize athletes based on their running patterns. To this end, we investigated the separability of movement patterns in two scenarios: (1) when movement patterns were expressed by variables of high relevance (derived via LRP), and (2) when movement patterns were defined by variables of low relevance. We expected the movement patterns of different individuals to separate well when expressed through high relevance variables, and to be indistinguishable from one another when expressed through variables of low relevance.

Specifically, we hypothesized that:

**Hypothesis** **1** **(H1).** *The movement patterns of different individuals would not overlap within a principal subspace, when movement patterns are defined by variables of high relevance*.

**Hypothesis** **2** **(H2).** *The movement patterns of different individuals would overlap within a principal subspace, when movement patterns are defined by variables of low relevance*.

To contribute to a deeper understanding of the unique and generic movement characteristics of elite runners, we then delineated the top 10% of variables that did and did not separate well between athletes.

## 2. Materials and Methods

This work resulted from a secondary analysis of data that was collected previously. For additional details on the protocol and the primary purpose of the analyzed data, the reader is referred to the original work [16].

### 2.1. Participants

Twenty healthy male triathletes (age: 31.65 ± 5.3 years; mass: 74.6 ± 7.3 kg; height: 182.9 ± 6.9 cm) participated in this study. All athletes trained to compete in the 2019 Ironman season and finish the distance in under 9 h and 30 min. Written informed consent was collected from all participants and approval for this research project was obtained from the ethics Commission of the Department of Psychology and Sports Science at the Goethe University in Frankfurt am Main, Germany (reference number: 2019-10).

### 2.2. Protocol

Overground running data were collected using the Xsens MVN Link System (Xsense Technologies B.V., Enschede, The Netherlands), an integrated full-body system comprised of 17 inertial measurement units that sampled at 240 Hz. The participants were equipped with the system while running alongside the river Main following a 4 km, flat, paved, and slightly curved path that was part of the 2019 Ironman European Championship course.

Prior to any testing, the participants changed into a Lycra suit (Xsens Technologies B.V., Enschede, The Netherlands), which facilitated the optimal and secure positioning of the individual sensors of the MVN Link System. Except for the sensors at the feet, which were taped to the participants’ own shoes, all the sensors were attached to the Lycra suit itself. The 17 sensors were located on the left and right foot, shank, thigh, hand, forearm, upper arm, shoulder, pelvis, torso, and head. A calibration procedure (including a neutral standing and walking trial) was performed as per the manufacturer’s recommendations [17] before the equipment—which was not worn by the athletes (i.e., laptop, power bank, etc.)—was placed in a backpack that was carried by an instructor, who remained close to the athlete during the test.

The testing protocol consisted of a 10-min ‘cold-run’ (CR), a 10-min ‘warm-run’ (WR), a 90-min cycling session, and a 4 km long ‘transition-run’ (TR). The CR was performed without any warmup exercise (no stretching, etc.), while the WR was performed immediately after the CR. Following the cycling session, the participants changed back into their running shoes, repeated the calibration procedure, and started their TR. For all the runs and participants, the specific speeds were set by the instructor, who was cycling ahead of the athlete to provide slipstream. The speeds were selected such that the athlete would finish the Ironman competition under 9:30 h.

### 2.3. Data Preparation

Using the MVN Analyze software with the internal ‘HD processing’ filter enabled, 18 three-dimensional joint angle trajectories (according to the standard of the international society of biomechanics) were extracted for CR, WR, and TR. The joint centers were located at the left and right ankle, knee, hip, wrist, elbow, shoulder, and the intersection of the L5S1, L4L3, L1T12, T9T8, T1C7 and C1head joints. The trajectories of each recording were inspected manually, and clear erroneous outliers were removed from further analysis. The remaining, clean, recordings were exported, and the subsequent analyses were performed in MATLAB (R2021a, The MathWorks Inc., Natick, MA, USA) via custom written routines.

Step cycles, defined as the period between two consecutive touch-down events, were parsed from the recordings. The touch-down events were identified using a two-step routine: first, the vertical positions of the foot (center of the calcaneus) and toe (metatarsal joint) were calculated, and the maxima of the resulting trajectory were determined. Within the period of two consecutive maxima, touch-down was identified as the first sample of either the foot or toe trajectory below a set threshold. A threshold of 2 cm above the local minimum of the respective (foot/toe) vertical position proved consistent. Similarly, take-off was identified as the last sample of either the foot or toe vertical position below the same threshold. Because foot and toe positions were considered, this routine guaranteed consistent event detection for forefoot and rearfoot strike patterns. Lab-internally, the quality of the routine was compared to an event detection by an instrumented treadmill using speeds ranging from 8 to 20 km/h for 20 individuals. We found the overall accuracy to be in the order of ± 10 ms.

The joint angles of isolated step cycles were time-normalized to 100 data points. Trunk angles in the frontal and transverse plane of the left side were mirrored to enable comparisons between the left and right side of the body. Additionally, the angles were labeled as belonging to the standing or swinging side, the standing side being the side of the standing leg and the swinging side being the side of the swinging leg. The abduction and rotation directions of the leg and arm angles were defined in anatomical dimensions. A single step cycle was characterized by 5400 data points (100 timepoints × 18 joint locations × 3 dimensions).

For one participant at a time, all step cycles were exposed to an outlier detection and scaling procedure: first, a principal component analysis was used to reduce the dimensionality of the step cycles. The loadings on the first three principal axes were then used to detect outliers following the procedure introduced by Kriegel et al. [18]. The remaining step cycles were then scaled by subtracting their respective mean, dividing by their respective standard deviation, and rescaling the result to range from −1 to 1. This scaling procedure was necessary to prepare the data for the neural network and layer-wise relevance propagation. All clean and scaled step cycles of all participants were arranged in a single data matrix of dimensionality 97,899 × 5400 (step cycles × data points).

### 2.4. Data Analysis

The deployed neural network consisted of three layers: one input, one hidden, and one output layer. This three-layer architecture was chosen as a single hidden layer is, reportedly, sufficient to learn most input–output relationships [19]. For each layer, the number of nodes was derived from the data: 5400 for the input layer (because a single step cycle was defined by 5400 variables), 10800 (2 × 5400) for the hidden layer, and 20 nodes for the output layer, one per participant. The hyperbolic tangent was used as an activation function for the hidden layer. The model was then trained on 200 randomly selected step cycles (10 per participant) that were derived exclusively from the pooled data of the CR and WR. The CR and WR data were pooled to provide a bigger selection of step cycles to choose from. During piloting, we explored how many step cycles per participant were necessary for the model to accurately recognize individuals. We found that a minimum of five step cycles per participant was needed to achieve a testing accuracy of above 99%. Specifically, we trained the network on an increasing number of randomly selected step cycles (1–15) from the training data set (i.e., CR and WR) and each time we evaluated the performance of the trained network on the testing data (i.e., TR). The training of the model was performed in batch sizes of 25 with an epoch limit of 1000, which were found to be viable trade-offs between training efficiency and model accuracy. The network’s performance was then determined by classifying each step cycle of the TR data and calculating the participant-wise accuracy using Equation (1), where, for a given participant p, n described the number of correctly assigned step cycles and N the total number of step cycles.

(1) $$Accuracy\;\left(p\right)=\frac{n_{p}}{N_{p}}*100$$

The fully trained neural network model was then used to determine the relevance scores that were key to the subsequent analyses. For each correctly classified step cycle (from the TR data), the corresponding relevance scores were calculated using the layer-wise relevance propagation toolbox by Lapuschkin et al. [20]. The 5400 relevance scores of a given step cycle represented the corresponding relevance pattern. All relevance patterns were smoothed, whereby the previous and subsequent points were weighted with 25%, and the current point with 50%. This smoothing process was repeated twice. Because the input data (i.e., step cycles) were collected in the time-domain, the neighboring values were dependent and represented related information. The applied smoothing process therefore, reduced fluctuations in the calculated relevance scores without affecting the general pattern. The weights for the smoothing process were chosen so that their sum equaled 1 and a repetitive application would mimic a Gaussian filter. The smoothed relevance patterns were averaged in a participant-wise fashion, rectified, and normalized to their respective maximum. This process allowed us to express the relevance of any given variable with respect to the most relevant variable within the entire pattern of a given participant. The overall average was then calculated, normalized to its respective maximum, and sorted in descending order, ranking variables in their importance for human identification.

The similarity between step cycles based on variables of high/low relevance was then determined in an iterative procedure. First, all the step cycles of the TR were reduced to only those variables of high/low relevance that were included in any given iteration. For instance, the first iteration used only the 10 most/least important variables. Both subsets of data (high/low) were then exposed to a principal component analysis and the projections of the step cycles onto the first three principal axes were determined. In this three-dimensional space, any step cycle was represented as a single point. All step cycles of one participant would, therefore, form a cloud of points in the principal subspace. For each participant, MATLAB’s inbuild function *alphashape* was used to create a bounding volume that captured all step cycles of the given participant. The overlap of a participant’s bounding volume with all the other bounding volumes was then determined and expressed as a percentage of the given participant’s total volume. This procedure was repeated, incrementing the number of variables that were used to express the step cycles by five at every iteration until all 5400 variables were included again.

## 3. Results

**Figure 1:**
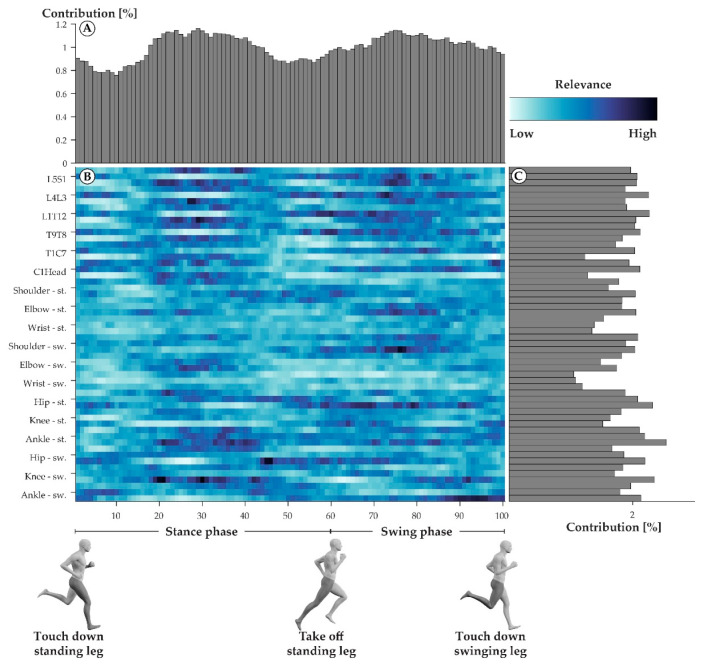
Averaged relevance scores per variable (**B**) and contributions of individual percentage points within a step cycle (**A**) and joint angle trajectories (**C**). In the center (**B**), darker colors indicate variables with high averaged relevance scores, while lighter colors indicate variables with low averaged relevance scores. The joint labels on the left-hand side of the heatmap each correspond to three rows, where the first row always describes the joint’s abduction, the second row the joint’s rotation, and the third row the joint’s flexion trajectories. The top part of the figure (**A**) shows the vertical summation of the heatmap, highlighting the contribution of each percent of a step cycle to the success of the model. The right part of the figure (**C**) depicts the horizontal summation of the heatmap, highlighting the contribution of each joint angle trajectory to the success of the model.

The neural network model that was trained on the CR and WR data to match step cycles to their respective athletes was 100% accurate when tested on the TR data. The averaged relevance scores that were derived from the neural network model varied greatly across all the variables of a step cycle (Figure 1B). For an accurate identification of individuals, every percentage point (Figure 1A) and every joint angle trajectory (Figure 1C) was needed. However, the variables from mid-stance (20–40%) and mid-swing (70–80%) were, on average, slightly more relevant. The variables describing the joint motion of the wrist of the swinging side were the least relevant for identifying the triathletes.

**Figure 2:**
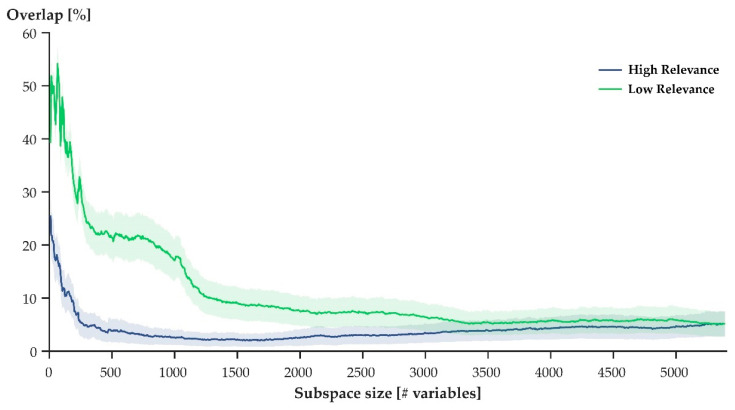
Average (± SEM) overlap of movement patterns in subspaces defined by the first three principal components derived from increasing numbers of variables with high (**blue**) and low (**green**) relevance. Overlap describes the intersecting volume of two point clouds that resulted from projecting movement patterns onto the first three principal axes for two participants. Using a pairwise comparison of all participants, the average was obtained.

When step cycles were defined through the variables with the lowest relevance scores (Figure 2: green), they overlapped substantially more than when step cycles were defined through the variables with the highest relevance scores (Figure 2: blue). For both low and high, increased subspace sizes resulted in reduced overlap. When the variables with high relevance scores were used to define step cycles, however, the decline in overlap occurred faster compared to when the variables of with low relevance scores were used to define the step cycles.

**Figure 3:**
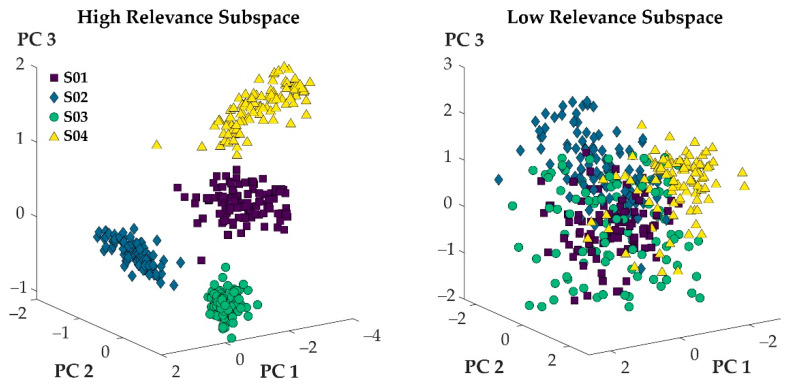
100 randomly selected steps from four participants represented in the subspace defined by the first three principal components of the 100 most relevant (**left**) and least relevant (**right**) variables.

Figure 3 visualizes the projection of step cycles onto the first three principal axes for 100 randomly selected step cycles from four participants. When the variables with high relevance scores were used to define step cycles, the resulting point clouds of the different participants separated well, and minimal overlap was observed (Figure 3: left). When the variables with low relevance scores were used to define the step cycles, the resulting point clouds did not separate, and maximal overlap was observed (Figure 3: right).

**Figure 4:**
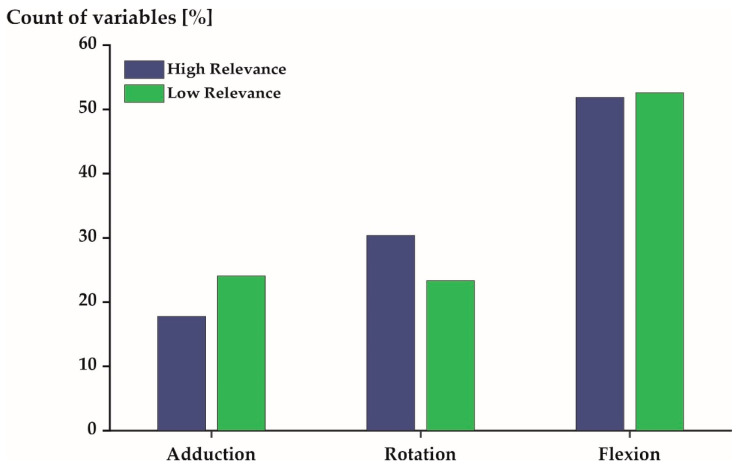
Distribution of the 540 most (**blue**) and least (**green**) relevant variables stratified by joint movement.

**Figure 5:**
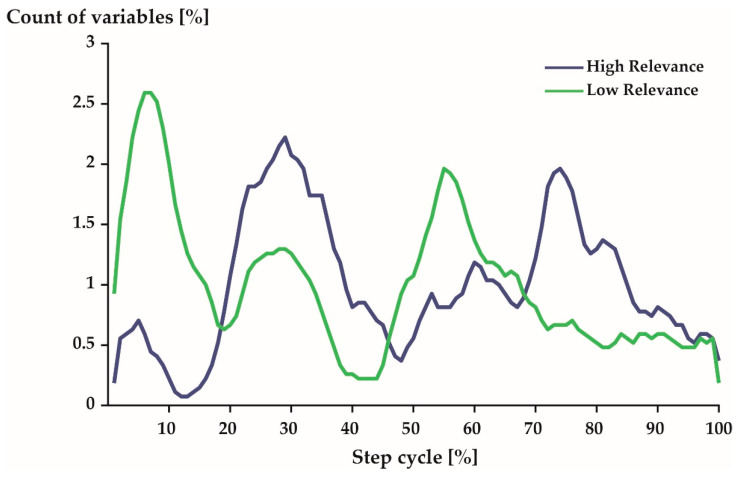
Distribution of the 540 most (**blue**) and least (**green**) variables over 100% of a step cycle. Note: A 5-point moving average filter was applied to smooth the represented curves. Further, 1%–60% represents stance phase and 61–100% represents swing phase. See Figure 1 for comparison.

**Figure 6:**
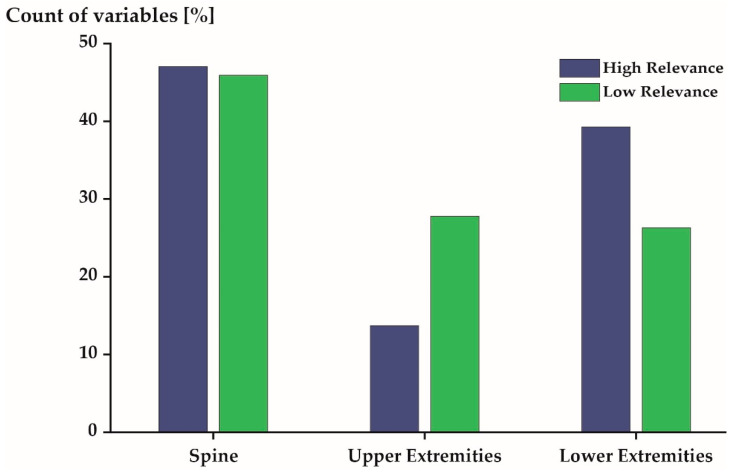
Distribution of the 540 most (**blue**) and least (**green**) relevant variables stratified by joint locations. Spine includes the L5S1, L4L3, L1T12, T9T8, T1C7, and C1 Head joints. Upper extremities includes the Shoulder-st., Shoulder-sw., Elbow-st., Elbow-sw., Wrist-st., and Wrist-sw. joints. Lower extremities includes the Hip-st., Hip-sw., Knee-st., Knee-sw., Ankle-st., and Ankle-sw. joints.

Figure 4, Figure 5 and Figure 6 profile the 540 variables (10% of all variables) that were, on average, most/least relevant in more detail. More than 50% of the 540 variables expressed flexion/extension movements in both the most and least relevant subspaces (Figure 4). The other movements (ab-/adduction and internal/external rotation) were described by 30% (or less) of the profiled variables.

With respect to time, the 540 most relevant variables were distributed predominantly around 29% and 74% of a step cycle (Figure 5: blue). The least relevant variables were most prominent during early stance phase (7%) just after touch-down and late stance phase (55%) right before take-off (Figure 5: green).

The 540 most relevant variables were predominantly derived from the joints located at the spine (47%) and the lower extremities (39%). Few variables (14%) were derived from the upper extremity joints (Figure 6: blue). The 540 least relevant variables were derived from the spine, the upper extremities, and the lower extremities, with 46%, 28%, and 26%, respectively (Figure 6: green).

## 4. Discussion

The observation that individuals move in their own unique manner has resulted in various works that have explored the identification of individuals based on their respective movement patterns [10,11,21,22,23]. Methodologies that utilize machine learning methods, such as support vector machines or artificial neural networks, generally report high accuracies when identifying individuals by their movement patterns [7,9,10,24]. However, not every aspect of a movement pattern is equally relevant to the identification of individuals. Some aspects (i.e., movement characteristics) were shown to be more important to a neural network than others [7], a finding supported by the results of this study (Figure 1).

Consequently, we explored the idea that any movement pattern might be a combination of unique (individual-specific) and generic (common to many individuals) movement characteristics. The unique aspects of a movement pattern may be encoded in those variables that are more relevant to a neural network when identifying individuals, while generic aspects may be encoded in those variables that are less relevant to the neural network. Specifically, we hypothesized that the movement patterns of individuals would not overlap (i.e., would be distinct) in a data subspace derived from variables that were highly relevant to identifying individuals based on their movement patterns (determined by layer-wise relevance propagation). Conversely, in a data subspace derived from variables that were identified as less relevant, the movement patterns of different individuals would overlap and be indistinguishable from one another.

In general, both hypotheses were supported when considering a select few participants (Figure 3). When variables of high relevance were used to express movement patterns, a projection of movement patterns onto the first three principal axes of the given data set showed no overlap between the movement patterns of different athletes (Figure 3: left). The projections of movement patterns that were expressed through variables of low relevance overlapped substantially (Figure 3: right). Averaging the results across the entire study population, however, showed that some overlap (although small) persisted when variables of high relevance defined movement patterns (Figure 2: blue). Ultimately, then, H1 needs to be rejected, while H2 was supported by the findings of this work.

A possible explanation for the rejection of H1 might be associated with the homogeneous nature (male Ironman competitors) of the study population: elite athletes adjust their movement patterns to be more efficient [25], as running economy is key to success in competition. Similarities between movement patterns that are optimized to be more economical, then, are to be expected when the margin of error in competition is vanishingly small. In fact, previous studies showed that certain kinematic variables (e.g., range of motion) are too unspecific to distinguish between individuals within a homogenous study population [26,27]. A second explanation for the residual similarities in movement patterns might be related to the fact that movement patterns were defined through joint angle trajectories only. When humans recognize friends and family members by means of their movement, they do so via visual cues that include information such as intent, motor effort, gender, and others [28,29,30]. For instance, Johansson [31] has shown that the emotional state of an individual can be identified when movements are presented as simple point lights. A movement pattern derived exclusively from joint angle trajectories, however, does not capture information about height, weight, and/or emotional states. Consequently, the ‘uniqueness’ of a movement pattern might be inherently limited if a movement is described exclusively by joint angle trajectories. Future research would have to show if more holistically defined movement patterns would result in less overlap. Given these considerations, it appears reasonable that our results did not show a perfect separation of movement patterns within subspaces defined by the most relevant variables.

While H2 was supported by our results, the maximal observed overlap did not surpass 60% (Figure 2: green), suggesting that some degree of ‘uniqueness’ remained within the variables that defined a movement pattern. This finding contrasts our initial speculation that the movement patterns of different athletes would be indistinguishable when variables of low relevance were used to define movement patterns. A possible explanation for the remaining ‘uniqueness’ within variables of low relevance might be the variability inherent to human movement [32,33]. Additionally, factors such as joint laxity, footwear, step length, anthropometrics, and others were not controlled for in this study and, consequently, it is likely that they contributed to some measure of ‘uniqueness’ within the studied joint kinematics. Given these considerations, the initial speculation that movement patterns would overlap perfectly needs to be revaluated: some degree of individuality should be expected, even within those variables that are least relevant for a neural network to identify individuals.

In summary, similarities between the movement patterns of different athletes were small when the movement patterns were reduced to those variables that were most relevant for a neural network to distinguish between athletes. Variables of high relevance, therefore, isolated movement characteristics that separated well between individuals and may be considered unique. Similarities between the movement patterns of different athletes were large when the movement patterns were expressed through those variables that were least relevant for a neural network to distinguish between athletes. Variables of low relevance, therefore, isolated movement characteristics that do not separate well between individuals and may be considered generic.

It is of interest to examine the unique and generic movement characteristics of the studied population in greater detail as they may have implications for health [8,9], security [10], and performance [11]. The unique movement characteristics of elite triathletes were predominantly sagittal plane movements (Figure 4) of the spine and lower extremities (Figure 6) during mid-stance and mid-swing (Figure 5). Conversely, the generic movement characteristics of elite triathletes were sagittal plane movements (Figure 4) of the spine (Figure 6) during early and late stance (Figure 5).

This contrasts with previous findings, which suggested that coronal and transverse plane movements are unique, while sagittal plane movements are more generic [7]. However, this comparison should be treated with caution as the cited work studied novice runners and a limited set of joint angles, while the present work investigated elite runners and a more extensive set of joint angles. Nonetheless, the predominance of sagittal plane movements within unique and generic movement characteristics remains striking. A potential explanation might be that postural control and running movements are mostly confined to the sagittal plane [34,35,36]. In this plane, a joint’s range of motion tends to be larger [37]; thus, similarities and dissimilarities in flexion/extension movements between individuals might simply be more common. That generic movement characteristics of elite runners were confined to the early and late stance phase appears reasonable when considering that the movements of recreational and elite runners differ within these periods [38,39]. Additionally, some running programs cue their participants to adjust their landing strategies [40], so that ‘overstride’ is reduced and longer flight times are promoted. Amongst others these are defining characteristics of elite runners [41]. Consequently, it seems reasonable that elite runners would express similarities during early and late stance, while individual movement strategies may vary during mid-stance and mid-swing. Finally, it is not surprising that both unique and generic movement characteristics were dominant in spinal motion. Both extremities (lower and upper) are connected via the trunk and, thus, spinal motion reflects upper and lower extremity motion. As a result, an individual’s unique movement strategy will be reflected in spinal motion just as much as a generic arm swing during running.

In summary, the unique and generic movement characteristics of elite runners are predominantly flexion/extension movements of the spine. However, unique strategies occur during mid-stance and mid-swing, while generic movements occur during early and late stance.

A limitation of this study was that only well-trained triathletes participated. This is important because triathletes have high training volumes in running, swimming, and cycling. It is unclear whether the presented results would generalize well to the broader population of runners. The running patterns of novice athletes and males and females, however, are known to be distinct enough to differentiate between them. Consequently, similar findings may be expected from different study populations. Further, the participants did not run at the same speed, and it is well accepted that running speed changes running patterns. While the running speed was self-selected, it was based on the participants’ finishing time for an Ironman competition, and all the participants were able to finish the competition in under 9 h and 30 min. The running speeds were therefore comparable across the participants and the small variation in speed was considered acceptable, as variations of 0.1 m per second can be observed in treadmill running as well. Finally, the relevance scores presented in this work were all dependent on the data the trained neural network received. They should therefore be treated with caution and a generalization of their importance should be avoided.

## 5. Conclusions

Human movement patterns are a composition of unique and generic movement characteristics. Unique characteristics include aspects that are specific to any given individual, while generic characteristics include aspects that are common to all individuals. For elite runners, unique movement characteristics were found to be predominantly sagittal plane movements of the spine and lower extremities during mid-stance and mid-swing. The generic movement characteristics were found to be sagittal plane movements of the spine during early and late stance.

## Data Availability

All underlying data and analysis routines that are necessary to replicate the results presented in this work can be found here: https://www.move-functional.at/downloads.php (accessed on 26 July 2021). The download requires a username (research) and a password (uni2o21@RUN).
